# Spectrum and Clinical Outcome of Motility Disorders on High-Resolution Esophageal Manometry: A Study From a Tertiary Center on Patients With Dysphagia in Pakistan

**DOI:** 10.7759/cureus.12088

**Published:** 2020-12-14

**Authors:** Hareem Rehman, Adeel Abid, Safia Awan, Farheen L Hashmi, Shahab Abid

**Affiliations:** 1 Medicine, Aga Khan Health Service, Karachi, PAK; 2 Internal Medicine, Aga Khan Health Service, Karachi, PAK; 3 Gastroenterology, Aga Khan University, Karachi, PAK

**Keywords:** dysphagia, manometry, achalasia

## Abstract

Objective

This study aimed to evaluate the outcome of high-resolution esophageal manometry (HRM) in the diagnosis of esophageal motility disorders in a Pakistani population. It also evaluates the outcomes of management of patients with esophageal dysphagia and no structural abnormality on endoscopy.

Methods

This is a cross-sectional study. Patients with symptoms of dysphagia with normal endoscopy were subjected to esophageal manometry and to barium swallow as a part of routine workup. Esophageal motility disorders diagnosed by HRM were compared to barium swallow findings. A follow-up of these patients was done after a one-year interval to evaluate improvement in their symptoms.

Results

A total of 202 patients underwent HRM. There were abnormal findings in 160 patients (79.2%) with achalasia being the most common diagnosis in 35.6% of the total patients. Out of 72 patients diagnosed to have achalasia on HRM, only 46 (32.6%) had similar findings on barium esophagram and this difference is statistically significant, p < 0.001). Among achalasia patients, laparoscopic surgery was performed in 22 (30.5%) patients with 59% patients reporting a good to excellent improvement (>50%) in their symptoms, balloon dilatations were done in 47 (65.27%) patients with a good to excellent improvement in symptoms in 55% patients. Only three patients (5.5%) were given botulinum toxin injections, and two of them had an improvement of >50% in their symptoms. Patients with motility disorders other than achalasia were treated with a combination of proton pump inhibitors (PPIs), calcium channel blockers and selective serotonin reuptake inhibitors (SSRIs).

Conclusion

Achalasia was the most common esophageal motility disorder in our population. HRM can diagnose significantly more patients with achalasia compared to barium swallow. Likewise, HRM was helpful in detecting other esophageal motility disorders and planning their management.

## Introduction

Esophageal dysphagia refers to a difficulty or an abnormality in swallowing food. Dysphagia could be with solids, liquids or both, and it could be progressive or intermittent. It is an important alarm symptom and warrants urgent exclusion of an organic cause via esophageal endoscopy. Dysphagia could be both structural and non-structural. The most prevalent obstructive etiologies leading to esophageal dysphagia are esophageal cancer, peptic strictures and eosinophilic esophagitis [1]. In non-obstructive dysphagia there is usually an absence of any endoscopic or radiologic evidence of a lesion that may lead to an obstruction of a bolus [2], and the symptoms are usually secondary to a motor dysfunction. The major esophageal motor disorders are achalasia, esophagogastric junction outflow obstruction, diffuse esophageal spasm, and absent peristalsis [1].

Achalasia and diffuse esophageal spasm are the most common esophageal motility disorders. In achalasia, there is a loss of inhibitory neurons in the myenteric (Auerbach’s) plexus which leads to a failure of relaxation of the lower esophageal sphincter (LES) with swallowing. Additionally, there is aperistalsis of the esophageal smooth muscles [3]. The presumptive diagnosis of achalasia is made by clinical symptoms, and then confirmed via esophagogastroduodenoscopy, barium swallow and manometry. Endoscopy is not a very sensitive test for identifying esophageal motor disorders and is mainly useful in excluding other causes such as malignancy. Barium swallow reveals a classic bird-beak appearance due to narrowing at the gastroesophageal (GE) junction and proximal dilation of the aperistaltic esophagus [4]. Recent studies have proven that high-resolution esophageal manometry (HRM) should be the diagnostic test of choice for primary esophageal dysmotility disorders [5].

Esophageal manometry assesses the motility by measuring the amplitude of the contractile events within the esophagus and its sphincters in relation to time [6]. HRM has better ability to diagnose achalasia and its variants, by using up to 36 pressure sensors placed 1 cm apart along the catheter. The Chicago classification categorized achalasia into three variants, based on HRM findings on the presence of peristalsis in the esophageal body and relaxation of the LES [7]. There is a dearth of research in the area of esophageal motility disorders in terms of their diagnosis and management. The present study is aimed to evaluate the outcome of HRM, its comparison with barium swallow in the management of such patients, and the outcomes of management.

## Materials and methods

This is a cross-sectional analysis of patients with symptoms of dysphagia with normal endoscopy who were subjected to esophageal manometry and to barium swallow as a part of routine workup. The study was conducted at the Aga Khan University Hospital in Karachi, Pakistan. A total of 202 patients, who presented to the outpatient gastroenterology clinic with complaints of dysphagia and had normal findings on endoscopy, were recruited during a period of June 2017 to May 2019. Their demographics such as age, gender, BMI and presenting symptoms were recorded. Patients were categorized according to the Chicago classification v3.0 [7]. Group 1 includes disorders with esophagogastric junction (EGJ) outflow obstruction, Group 2 includes major disorders of peristalsis (Jackhammer, distal esophageal spasm [DES] and absent contractility), Group 3 includes minor disorders of peristalsis and Group 4 is normal. Disorders with EGJ outflow obstruction include the three subtypes of achalasia. Weak peristalsis with small peristaltic defects and weak peristalsis with large peristaltic defects is combined into a collective disorder called ineffective esophageal motility (IEM), within group 3 of the Chicago classification. Findings of esophageal manometry were compared to barium swallow findings.

The frequencies (n%) of various esophageal motility disorders in our study population was tabulated and data was analyzed using SPSS 19.0 (IBM Corp., Armonk, NY). The different modalities of treatment were compared using the chi square test. All p-values were two sided and a value of <0.05 was considered significant. These patients were treated as standard of care based upon their symptoms and underlying diagnosis.

A follow-up of these patients was done at one-year time to evaluate improvement in their symptoms. Patients’ symptoms improvement was categorized into three groups; on the basis of >50% improvement of symptoms as ‘good to excellent’ response, >25% but <50% improvement of symptoms as ‘fair to good’ response or <25% improvement/no improvement/recurrence of symptoms.

This study protocol was approved by the Ethical Review Committee, Aga Khan University Hospital Karachi. The reference number is 2020-5244-14782.

## Results

A total of 202 patients were identified and underwent high resolution esophageal manometry. The demographics of the study population are included in Table 1. These patients had normal findings on esophageal endoscopy. The HRM findings were normal in 42 patients (20.8%). Achalasia was identified in 72 patients (35.6%) and hence was the most common esophageal motility disorder in our study population. The second most common disorder was diffuse esophageal spasm, present in 28 patients (13.0%). The other less common disorders diagnosed via high-resolution manometry (HRM) included ‘weak peristalsis with large peristaltic defects’ (7.9%), ‘EGJ outflow obstruction’ (5%), ‘weak peristalsis with small peristaltic defects’ (4%), ‘absent peristalsis’ (4%) and ‘Jackhammer’ (2%). Fourteen patients had other non-specified (rapid contraction) esophageal motility disorders (Table 2).

**Table 1:** Characteristic of study population (n = 202).

Age		47.8 ± 17.2
BMI		23.9 ± 6.4
Gender [N (%)]	Female	102 (51)
	Male	100 (49)
Symptoms [N (%)]	Chest pain with dysphagia	14 (6.93)
	Dysphagia	152 (75.24)
	Vomiting with dysphagia	36 (17.83)

**Table 2:** Spectrum of esophageal motility disorders on HRM. HRM: High-resolution manometry; EGJ: Esophagogastric junction.

DISEASE	FREQUENCY	PERCENT
Achalasia	72	35.6
Distal esophageal spasm	28	13.9
Weak peristalsis with large peristaltic defects	16	7.9
Others (not specified, rapid contraction)	14	6.9
EGJ outflow obstruction	10	5.0
Absent peristalsis	8	4.0
Weak peristalsis with small peristaltic defects	8	4.0
Jackhammer	4	2.0
Normal	42	20.8

Out of the 72 patients who were diagnosed with achalasia on HRM only 46 had consistent findings in their barium swallow study (p < 0.001), whereas 12 patients with achalasia diagnosed by HRM had normal barium swallow findings.

Only a minority of patients, 22 out of a total of 72 diagnosed with achalasia, underwent laparoscopic surgery. Out of which 59% patients reported a good to excellent improvement (>50%) in their symptoms, while 27% reported a fair to good improvement (>25% and <50%) and 13% had no improvement (<25%)/relapse of symptoms. Balloon dilatation was the most popular treatment modality. A total of 47 patients got balloon dilatation done. A 30-mm dilator was used and dilatation was performed once only. The outcome was good to excellent improvement in symptoms in 55% patients, followed by fair to good improvement in 21%, and no improvement or relapse in 24%. Overall, only three patients opted for BOTOX injections, and within this sub-group, two patients had an improvement of more than 50% while one patient had an improvement of more than 25% but less than 50% in their symptoms. Responses to various treatment modalities for achalasia are shown in Table 3.

**Table 3:** Outcome of treatment of esophageal motility disorders. SSRI: Selective serotonin reuptake inhibitor; PPI: Proton pump inhibitor.

	Modality of treatment (n)	Good to excellent >50% improvement N (%)	Fair to good >25% but <50% improvement N (%)	No improvement or relapse <25% improvement N (%)	p-value
Achalasia	Surgery (22)	13 (59)	6 (27)	3 (13)	<0.001
	Balloon dilatation (47)	26 (55)	10 (21)	11 (24)	
	Botulinum toxin (3)	2 (66.7)	1 (33.3)	0	
Motility disorder other than achalasia	PPIs, calcium channel blockers, SSRIs (88)	24 (27%)	13 (15%)	51 (58%)	<0.001

A total of 88 patients who had motility disorders other than achalasia were treated with a combination of various treatment modalities, which included proton pump inhibitors, calcium channel blockers, and selective serotonin receptors blockers. Among these patients, significant resolution of symptoms was observed in 24/88 (27%) of patients on medications while 13/88 (15%) had partial response. However, no effect was seen in 51/88 (58%) patients. One patient with Jackhammer esophagus had a good response to per oral esophagomyotomy while other three were treated by smooth muscle relaxants and botulinum toxin with unsatisfactory improvement.

## Discussion

This study provides data of a large cohort of patients with esophageal motility disorders diagnosed using HRM and classified according to the latest Chicago classification v3.0. This is the first study from Pakistan that reports the spectrum of esophageal motility disorders, compares barium esophagram and HRM for diagnosis of achalasia, and identifies outcomes of patients with different treatment strategies, within our population.

This cross-sectional analysis, conducted on 202 patients, provides data on the spectrum of esophageal motility disorders in Pakistani population. The most common disorder in our study is achalasia, followed by weak peristalsis with large peristaltic defects. This is in concordance with a study conducted by Yeh et al., in the Taiwanese population [8], and in a study by Burgess and Wyeth [9]. Both recorded achalasia as the most common diagnosis, followed by ineffective esophageal motility. Abbas et al. also reported similar finding in a study conducted in Sudan [10]. However, in Chinese and North Indian population, ineffective esophageal motility disorder was found to be more common than achalasia [11-12]. The spectrum of disorders vary in different research groups based on different race, ethnicities, dietary habits and also selection of patients. Symptoms may also vary amongst patients. Most of our patients presented with an isolated complaint of dysphagia, while the remaining had dysphagia accompanied with either regurgitation/vomiting or with retrosternal chest pain. Normal HRM findings in one-fifth patients despite the presence of symptoms, could be suggestive of functional dysphagia, and it is advisable to get a psychiatric evaluation to rule out the possibility of somatization disorder.

Despite the fact that manometry is the gold standard for diagnosis of achalasia, barium swallow has been found to be a reliable test for its diagnosis by a few studies [13,14]. In our study, the sensitivity of barium esophagram for the diagnosis of achalasia was 63.9%. It is important to note that in 19.4% patients diagnosed with achalasia based on HRM findings, the barium study reported the presence of some other non-specific motility disorders. These findings were consistent with studies performed by El-Takli et al., in which the diagnosis of 42% patients with achalasia was missed by barium and by Schima et al., in which barium swallow correctly identified 58% of the cases diagnosed monometrically [4-15]. The low sensitivity of barium esophagram was attributed to the absence of the characteristic radiographic findings in most of the cases [4]. Good concordance between barium and manometry for the diagnosis of achalasia was also reported by Anumandla et al. [16]. Our radiographic sensitivity was relatively higher compared to some other studies such as O’Rourke et al. reported a very low sensitivity of barium swallow (29%) for diagnosis of achalasia [17]. Whereas Howard et al. reported that radiographic findings were consistent with the manometric diagnosis of achalasia in 45% patients [18]. Most of these findings were not in concordance with a study conducted by Ott et al., that had a radiographic sensitivity of 0.95 for achalasia in 172 patients presenting with dysphagia [19]. However, such a high radiographic sensitivity has never been reported again in subsequent studies.

Our data suggests that HRM should be performed in all patients presenting with normal esophagoscopy and persistent symptoms of dysphagia with or without vomiting or regurgitation, regardless of the results of other investigations. Since the radiographic sensitivity for the diagnosis of achalasia is not very high, it appeared to be an inferior screening tool and there is a high probability of missing cases if barium alone is preferred over HRM. There is some evidence that raising awareness amongst radiographers of the diagnostic features of achalasia on barium swallow might enhance the diagnostic power of the study [4]. Also, HRM diagnoses dysmotility much earlier in the disease process compared to barium swallow, which contributes to the low radiographic sensitivity.

Our study also evaluated the long-term outcomes of patients who underwent different treatment modalities. In achalasia the approach to management is directed towards relieving the LES pressure, which could be achieved by surgery, botulinum toxin, pneumatic dilatation, or per oral esophageal myotomy (POEM). Medical therapies that involve muscle relaxants such as calcium channel blockers and nitrates have been found to have a low efficacy and hence are not included in the popular management strategies [14].

Pneumatic balloon dilatations and surgery have been proven to be equally efficacious and more effective than BOTOX injections [20]. Even in our study there were comparable findings of good to excellent improvement after surgery and pneumatic dilatation (59% and 55%, respectively). Hence, botulin toxin therapy should be considered only when surgery and pneumatic dilatation are not possible options. Despite yielding similar improvement results, pneumatic dilatation has a higher rate of recurrence of symptoms compared to surgery. In our study, pneumatic dilatation was done in twice the number of patients compared to laparoscopic surgeries and had a higher rate of either no improvement or a relapse compared to surgery (24% vs 13%). Hence, repeated dilatations may be required. A relatively newer procedure, POEM, which was popularized by Inoue et al. in 2008, has proven to have an excellent short-term outcome for achalasia [21]. It is a form of natural orifice transluminal endoscopic surgery (NOTES) and is less invasive compared to laparoscopic Heller myotomy. By creating a submucosal tunnel, endoscopic access to the mediastinum is established through the walls of the esophagus, rather than through skin incisions in the traditional laparoscopic method [14]. Despite having promising short-term results, long-term prospective studies are required for establishing safety and durability of the procedure in long term [22]. EndoFLIP can be used to measure the EGJ distensibility intraoperatively and is a useful tool in ensuring that adequate myotomy is performed. This technique involves the use of a balloon electrode and functional imaging luminal probe for the measurement of the diameter of the esophagogastric junction [22]. Despite being a useful modality in predicting outcome of surgical procedures for achalasia, it is still a relatively newer procedure and not very widely used. Unfortunately, both POEM and EndoFLIP are not available in our institute.

This study has various strengths. To the best of our knowledge, this is the largest study conducted in Pakistan that reports the prevalence of several esophageal motility disorders, evaluates the diagnostic significance of barium swallow in comparison to esophageal manometry and also reports the long-term results of different treatment approaches to achalasia. One of the limitations of the study is that the outcome of treatment was only measured subjectively, in terms of improvement of symptoms, as recorded by the patients. We did not use any objective measure such as LES pressure, time barium esophagram or the Eckardt score for an objective measure of the clinical outcome. Additionally, only a single center was included leading to an increased risk of selection bias. Our center, however, is one of the few centers with a very good set up of gastrointestinal physiological testing including esophageal manometry.

## Conclusions

In conclusion, using the Chicago v3.0 classification, achalasia is the most common esophageal motility disorder diagnosed in patients with dysphagia in our study. Barium swallow can miss quite a few cases of achalasia diagnosed with high resolution esophageal manometry. Outcome of pneumatic dilatation was like surgical intervention for achalasia patients. Esophageal motility disorders other than achalasia by large have unsatisfactory outcome to medical management.
